# Drug Delivery Systems for Respiratory Diseases: Insights into the Therapeutic Innovations for Pulmonary Administration

**DOI:** 10.3390/pharmaceutics17050539

**Published:** 2025-04-22

**Authors:** Mershen Govender, Yahya E. Choonara

**Affiliations:** 1 Wits Advanced Drug Delivery Platform Research Unit, Department of Pharmacy and Pharmacology, School of Therapeutic Sciences, Faculty of Health Sciences, University of the Witwatersrand, 7 York Road, Parktown, Johannesburg 2193, South Africa; 2 Wits Infectious Diseases and Oncology Research Institute, Faculty of Health Sciences, University of the Witwatersrand, Johannesburg 2193, South Africa

## 1. Introduction

Respiratory issues, such as asthma, chronic obstructive pulmonary disease (COPD), pulmonary fibrosis, pulmonary hypertension, and respiratory infections, are amongst the most common and debilitating medical conditions experienced worldwide, affecting millions of patients annually [1,2,3]. These diseases significantly influence patients’ quality of life, contributing to high morbidity and mortality rates and significant treatments costs [4]. The effective management of these respiratory disorders relies not only on the availability of effective drug molecules, but also on the ability to deliver these drugs efficiently to the target site in the respiratory system, thereby improving treatment outcomes and decreasing adverse effects [5,6].

Respiratory diseases typically affect the lungs, airways, and other components of the respiratory tract, with treatments primarily aiming to reduce inflammation, open airways, or overcome infections. Traditional administration routes, such as oral or intravenous delivery, however, often result in limited drug concentrations reaching the respiratory system with the potential for higher systemic exposure, leading to potential side effects [7,8]. Pulmonary drug delivery for the treatment of respiratory ailments is an effective and non-invasive route that offers several advantages over these traditional administration pathways, and the large absorptive surface area of the lungs, presence of thin alveolar epithelium, and extensive vascularization allow for both localized and systemic delivery of bioactives [9,10]. The delivery of drugs to the various parts of the respiratory system is, however, impacted by its complex anatomy and natural defence mechanisms, such as mucociliary clearance, native immunity and enzymatic barriers [11,12,13]. Furthermore, the use of conventional inhalation therapies, such as metered-dose inhalers, dry powder inhalers, and nebulizers, despite their demonstrable clinical value, are often associated with poor drug solubility, rapid clearance, low deposition efficiency, inadequate targeting, and limited control over drug release [14].

Advanced pharmaceutical systems which allow for enhanced delivery of loaded bioactives to the specific areas of the lungs represent potential solutions to this challenge. These platforms include, amongst others, micro- and nano-systems, such as polymeric micro- and nanoparticles, solid lipid nanoparticles, liposomes, and micelles, which are delivered via inhalers, nebulizers, or through the intrathecal or endotracheopulmonary routes [11,15,16]. Micro- and nano-systems, which are defined by their dimension, offer multiple advantages, including enhanced solubility of poorly water-soluble drugs, protection of labile biomolecules, prolonged residence in the lungs, and the potential for controlled and modified drug release. Their aerodynamic properties also allow for deep penetration of the alveolar region of the lungs, with surface modifications, such as PEGylation or ligand conjugation, improving targeting and retention, while rapid clearance or immune activation are avoided [17,18,19]. Therefore, it is imperative to design these advanced systems in such a way that the delivered bioactive reaches the required area within the respiratory system, while simultaneously overcoming the body’s natural defence mechanisms and reducing the associated adverse effects [20,21].

Furthermore, while the development of novel advanced drug delivery systems for pulmonary administration has shown positive results, researchers have also investigated the optimization of traditional delivery devices for improved delivery of approved, regulated drug molecules and products [22,23]. Such studies are vital in the field of drug delivery, as they lead to effective utilization of resources, thereby improving treatment outcomes, while limiting the costs and development time of products.

## 2. Overview of the Published Articles

This Special Issue, which comprises seven research articles and one review paper, offers insights into the recent advancements in drug delivery for the treatment of respiratory diseases with the aim of enhancing therapeutic efficacy and decreasing adverse effects. This is exemplified in the design of paclitaxel (PTX)-loaded mesoporous polydopamine nano-bowls for the potential treatment of non-small-cell lung carcinoma (NSCLC) by Ngema et al. (Contribution 1). In this study, the bowl-shaped nanocarrier with connecting mesoporous channels and a central hollow cavity displayed a mean diameter of 200.4 ± 5.2 nm and a surface charge of −39.2 ± 1.3 mV, ideal for deposition in the alveolar region of the lung, with an entrapment efficiency of 95.7% and release of 85.1% under simulated tumour microenvironment conditions (pH 5.9) after 48 h. Cell proliferation studies further noted that the developed nano-bowls significantly suppressed A549 lung cancer cell proliferation at 48 and 72 h (cell viability of 14.0% and 9.3%, respectively), at a concentration of 100 μg/mL, thereby highlighting the potential of the platform for NSCLC treatment. Along these lines, glycerosomes (glycerol-developed nanovesicles) were also developed as an innovative strategy for the pulmonary delivery of itraconazole, via intratracheal administration, for the potential treatment of lung cancer by Aati et al. (Contribution 2). The researchers concluded that the drug-loaded hyaluronic acid-functionalized glycerosomes were 210.23 ± 6.43 nm in size, had a zeta potential of 41.06 ± 2.62 mV, an entrapment efficiency of 73.65 ± 1.76% and a far lower IC_50_ of 13.03 ± 0.2 μg/mL on the A549 cell line, when compared to an itraconazole suspension (28.14 ± 1.6 μg/mL). The glycerosomes also displayed a significantly higher accumulation (3.6x) and residence time (14 h vs. 6 h) in lung tissue when compared to the control suspension, thereby displaying significant promise for lung cancer interventions. The development of microsystems can also improve the treatment of respiratory inflammatory conditions, such as pulmonary fibrosis, a significant complication of COVID-19 infections. This was the focus of the study by Rzewińska et al., who prepared jet-milled and spray-dried microparticles with a d90 of 7.75 μm and 8.13 μm, respectively (Contribution 3). The microparticles, which can be delivered via dry powder inhalation, were loaded with CPL409116, a selective dual Janus kinase inhibitor and rho-associated protein kinase inhibitor. The study noted no significant difference in the dissolution rate of the microparticles prepared using the two techniques, with jet milling proposed as the preferable formulation process due to its lower cost, improved efficiency, and increased process understanding. These preliminary results highlight the platform’s potential to treat inflammatory respiratory conditions, though further toxicity, pre-clinical, and clinical studies are required to assess the microparticles for improved therapeutic effectiveness.

The optimization of delivery devices such as inhalers and nebulizers or the use of alternate delivery processes also improves the delivery of existing drug molecules and formulations for potential therapeutic benefits. Bruneau et al. focused on the use of vaping technology to deliver an approved drug, beclomethasone dipropionate, compared to jet nebulization (Contribution 4). The results of this study indicate that vaping devices are a potential alternative to nebulizers for the administration of beclomethasone dipropionate, with an equivalent dose delivered between the two devices. The vaping device produced smaller particles compared to the nebulizer (1.56 ± 0.05 μm vs. 2.30 ± 0.19 μm), allowing for disposition in lower areas of the lungs. Following this trend of utilizing nebulization for enhanced drug delivery, Otto and co-workers (Contribution 5), evaluated the potential of inspiration-synchronized vibrating mesh nebulizers and continuous vibrating mesh nebulizers for the delivery of aerosolized iloprost using an in vitro developed model of mechanically ventilated adults. The study protocol specifically assessed volume- and pressure-controlled continuous mandatory ventilation and their drug deposition rate and nebulization time. The drug disposition rate was comparable between the two nebulizer types during volume-controlled continuous mandatory ventilation, with pressure-controlled continuous mandatory ventilation achieving a result that was 10.9% lower in the continuous vibrating mesh nebulizer. This study provided insight into the use of differing nebulizer types and ventilation modes on drug delivery with future development work and clinical studies possible for the optimization of nebulized iloprost. The use of high-flow nasal oxygen therapy for the treatment of respiratory conditions has become increasingly prevalent in both the hospital and home settings, and was a key component in the treatment of severe COVID-19. Mac Giolla Eain and MacLoughlin focused on the effectiveness of in-line aerosol therapy via a nasal cannula using simulated adult and pediatric models with healthy, obstructive and restrictive lung types (Contribution 6). They concluded that increases in supplemental gas flow rates resulted in a decrease in aerosol delivery, regardless of the lung type, while large tidal volumes and extended inspiratory phases resulted in the greatest aerosol delivery. From this study it was determined that gas flow to inspiratory flow ratios of 0.29–0.5 were optimal for aerosol delivery and that controlling gas-flow rates is imperative. In a further effort to provide guidance on inhalation-based drug delivery, Lopez-Campos et al. (Contribution 7) undertook a systematic search of all doses used in devices for inhalation therapy on the Spanish Ministry of Health Billing List. The study identified 90 unique products in various drug classes, including long-acting bronchodilators (and its combinations) and corticosteroids, with differing information provided regarding the metered and delivered doses and the salt of the drug used. This study provides important information on the state of current products for respiratory conditions and offers guidance for clinicians regarding the importance of variable dosing in treatment interventions.

The final contribution to this Special Issue, which was research conducted by Sosnowski (Contribution 8), provided a review of pharmaceutical aerosols and their roles in drug disposition within the respiratory system, including the factors affecting aerosol effectiveness and the technical aspects of overcoming physiological barriers to pulmonary delivery and the key considerations for effective drug targeting. The specific devices used in the pulmonary delivery of aerosols were also reviewed in this contribution, which included a detailed discussion of the methods used to improve delivery, such as smart inhaling devices and systems with built-in AI algorithms. This review provides a comprehensive evaluation of the pharmaceutical systems and devices used to deliver aerosols to the respiratory system and is an excellent reference material for researchers in this field.

## 3. Conclusions and Future Directions

The development and evaluation of drug delivery systems, and the optimization of existing devices for the treatment of respiratory conditions, have advanced the use of such interventions to potentially offer enhanced therapeutic outcomes and decreased adverse effects compared with conventional treatments. With the rapid progression of research in this field, researchers are currently focusing on stimuli-response platforms, targeted formulations, theranostic systems, formulations that incorporate exosomes, three-dimensional (3D)-printed particles and nano-systems for the delivery of messenger ribonucleic acid (mRNA) and gene therapy. While diverse, the aim of these research endeavours is still aligned with the development of efficient, effective therapeutic interventions for the drug delivery and improved treatment outcomes of conditions affecting the complex respiratory system.

## Abbreviations

The following abbreviations are used in this manuscript:

3D	Three-dimensional.
COPD	Chronic obstructive pulmonary disease.
mRNA	Messenger ribonucleic acid.
NSCLC	Non-small-cell lung carcinoma.
PTX	Paclitaxel.
